# *In situ* polymerization of graphene-polyaniline@polyimide composite films with high EMI shielding and electrical properties†

**DOI:** 10.1039/c9ra08026k

**Published:** 2020-01-13

**Authors:** Kui Cheng, Haoliang Li, Mohan Zhu, Hanxun Qiu, Junhe Yang

**Affiliations:** School of Material Science and Engineering, University of Shanghai for Science and Technology Shanghai 200093 China lihl1989@outlook.com jhyang@usst.edu.cn

## Abstract

With the increasing demands of the electronics industry, electromagnetic interference (EMI) shielding has become a critical issue that severely restricts the application of devices. In this work, we have proposed a “non-covalent welding” method to fabricate graphene-polyaniline (Gr-PANI) composite fillers. The Gr sheets are welded with PANI *via* π–π non-covalent interactions. Furthermore, a flexible polyimide (PI) composite film with superior EMI shielding effectiveness is prepared by *in situ* polymerization. The 40% content of Gr-PANI_10:1_ (the mass ratio of Gr to PANI is 10 : 1) shows a superior electrical conductivity (*σ*) as high as 2.1 ± 0.1 S cm^−1^, 1.45 times higher than that of Gr@PI film at the same loading. Moreover, the total shielding effectiveness (SE_T_) of EMI of the Gr-PANI_10:1_@PI reaches ∼21.3 dB and an extremely high specific shielding effectiveness value (SSE) of 4096.2 dB cm^2^ g^−1^ is achieved. Such a “non-covalent welding” approach provides a facile strategy to prepare high-performance PI-based materials for efficient EMI shielding.

## Abstract 2

With the increasing demands of the electronics industry, electromagnetic interference (EMI) shielding has become a critical issue that severely restricts the application of devices.

## Graphical abstract

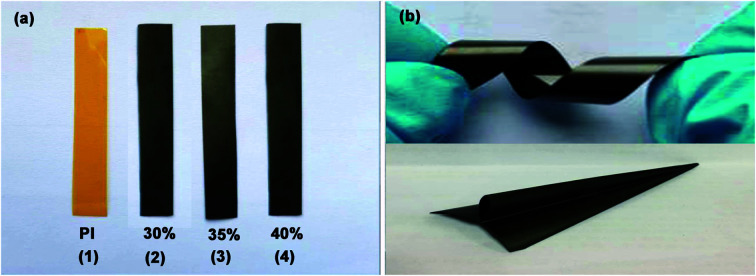

## 1. Introduction

With the rapid development of communication systems and electronic devices, electromagnetic interference (EMI) has become a serious problem as it not only interrupts the operation of electronic items, but also threatens people's health.^1,2^ The traditional EMI shielding materials made by metal show several limitations including high density, poor flexibility, and undesirable susceptibility to corrosion.^3^ Therefore, it is essential to develop novel materials with low density, high flexibility and high shielding effectiveness for EMI shielding.

PI, as a kind of high-performance engineering polymer, has been widely used in electronic industries as a promising polymer matrix for EMI shielding because of its superior flexibility, excellent thermal stability as well as high wear resistance.^4,5^ Great efforts have been taken to introduce Gr sheets into PI matrix to fabricate the composite film with high electrical conductivity and excellent EMI shielding property. Recently, carbon materials, such as carbon nanotubes (CNT),^6,7^ electrical carbon black,^8^ carbon fibers,^9^ and graphene^10^ have been explored and mixed with polymers as conductive fillers to fabricate lightweight and high shielding effectiveness polymer-based materials.

Among these potential carbon-based fillers, graphene, due to its outstanding electrical conductivity, has become a promising candidate as electrical filler in polymer.^11^ Feng and coworkers fabricated rGO@PANI/PI films *via in situ* polymerization. And the dielectric properties and thermal stability increased significantly.^12^ Ma *et al.* prepared rGO–poly(ethylene glycol) (PEG)/PI with chemically reduced method.^13^ However, it is very difficult to uniformly disperse rGO sheets in organic solvent owing to van der Waals force and the π–π stacking interaction between rGO sheets. Meanwhile, the interaction between the rGO sheets and PI matrix is relatively weak, leading to the inferior mechanical properties of PI/rGO film. In this regard, the thermal reduction of GO has advantages in mild reaction conditions and less deterioration of GO compared to chemical reduction of GO. A lightweight porous PI/rGO film was obtained using non-solvent induced phase separation (NIPS) process.^14^ They found that the PI/rGO film exhibited electrical conductivity of 1.5 × 10^−4^ S cm^−1^, corresponding to a SSE value of 693 dB cm^2^ g^−1^. The GO reduced through thermal reduction still maintains some oxygen functional groups, contributing to construct strong interfacial interaction with PI matrix. Therefore, these rGO@PI films exhibit the better mechanical properties. Nevertheless, these composite films reduced at high temperature have poor electrical conductivity limiting the improvement of electrical property and EMI shielding performance. An effective way to improve the dispersibility of Gr is needed to enhance the performance for EMI shielding and electrical conductivity.

In this work, we propose a “non-covalent welding” method to fabricate Gr-PANI filler to improve the electrical conductivity, mechanical and EMI shielding properties of the PI composite film. PANI, as a solder, not only successfully connects adjacent Gr sheets to provide the pathway for electron transportation, but also effectively prevents the stacking of Gr sheets *via* π–π non-covalent bonding. The Gr-PANI@PI film prepared through *in situ* polymerization, contributing to disperse Gr sheets homogenously in the PI matrix. The composite film displays superior mechanical properties (∼56.8 MPa), electrical conductivity (2.1 ± 0.1 S cm^−1^), EMI shielding performance (∼21.3 dB) and an extremely high SSE value of 4096.2 dB cm^2^ g^−1^ with a thickness of only 0.04 mm. The reported “non-covalent welding” conception is also an effective technique to prepare polymer materials with Gr sheets in other fields.

## 2. Experimental

### 2.1. Materials

Exfoliated graphene power, pyromellitic dianhydride (PMDA) and 4,4′-diaminodiphenyl ether (ODA) are purchased from Aladdin (Shanghai, China). Hydrochloric acid (HCl), aniline (AN), *N*-methylpyrrolidone (NMP), ferric chloride hexahydrate (FeCl_3_·6H_2_O) and ethanol were all purchased from National Pharmaceutical Group Chemical Co., Ltd.

### 2.2. Preparation of Gr-PANI composite filler

**Scheme 1:**
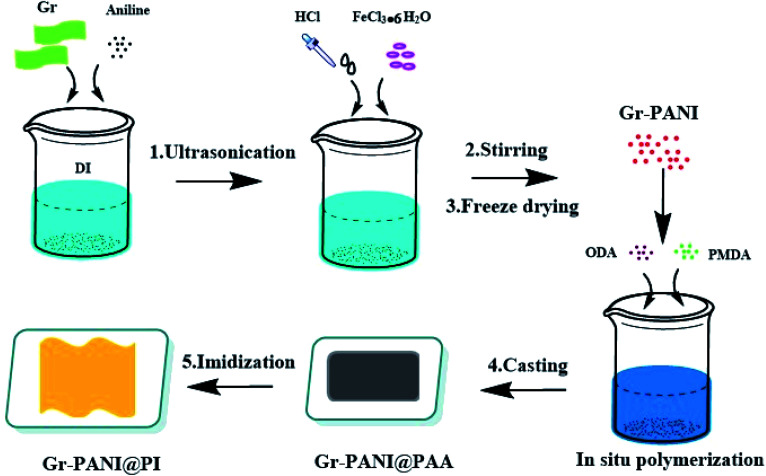
The fabrication procedure for Gr-PANI@PI composite film.

The preparation of Gr-PANI@PI composite films was illustrated in Scheme 1. Concretely, 1 g Gr and 0.1 g AN were dispersed in 100 mL of 1 mol L^−1^ HCl under ultrasonication. Then, the solution of FeCl_3_·6H_2_O (0.1458 g, 0.54 mmol) was rapidly transferred to the above solution containing AN and Gr. Next, the mixture was magnetically stirred for 4 h in an ice-water bath. After that, the Gr-PANI composite filler was obtained by filtration, washed with deionized water and methanol until the washing solution was completely colorless. In this process, free PANI particles and unreacted substance were separated and removed. Finally, the mixture was freeze-dried for 24 h. The as-obtained was named as Gr-PANI_10:1_(mass ratio of Gr to AN was 10 : 1). The Gr-PANI_15:1_ and Gr-PANI_5:1_ composite filler were prepared in the same manner.

### 2.3. *In situ* synthesis of Gr-PANI_10:1_@PI composite film

The fabrication process of Gr-PANI_10:1_@PI composite film involved three steps. First, 1.25 g Gr-PANI_10:1_ was dispersed in anhydrous NMP (15.33 mL) by ultrasonication (1 h) at ambient temperature in order to homogeneously disperse Gr-PANI_10:1_ in the solution. Then, ODA (2 g, 10 mmol) and PMDA (2.167 g, 9.94 mmol) were added into the solution under a nitrogen atmosphere. After continuous stirring at 25 °C for 2 h, the homogeneous and viscous Gr-PANI_10:1_@PAA (polyamic acid, the precursor of polyimide) solution was obtained. The mixture was bar-coated on a clean glass dish and treated at 140 °C for 1 h, and 350 °C for 2 h. The as-obtained composite film was coded as *x*%Gr-PANI_10:1_@PI, where *x* was the weight percentage of the Gr-PANI filler to the matrix. The 30%Gr-PANI_10:1_@PI obtained had a thickness of 40 μm. The 35%Gr-PANI_10:1_@PI and 40%Gr-PANI_10:1_@PI films were prepared in the same manner. In the same manner, we fabricated the *x*%Gr@PI (without PANI) composite film, where *x* represented the weight percentage of Gr filler to the matrix.

### 2.4. Characterization

Fourier transform infrared spectroscopy (FTIR, P.E. Spectrum 100, USA) was collected on a spectrum from 400 to 4000 cm^−1^ at a resolution of 2 cm^−1^ and for an accumulation of 8 times. Raman spectra (Horiba, LabRAM HR Evolution, France) were collected from 200 to 3000 cm^−1^ with a laser length at 532 nm. X-ray photoelectron spectra (XPS, Physical Electronics PHI 5000C ESCA) were performed to acquire the chemical bonding and the valence states of Gr and Gr-PANI with a monochromatized Al Kα X-ray source (1486.71 eV). X-ray diffraction (XRD, Bruker D8 ADVANCE, USA) patterns were conducted using nickel-filtered Cu Kα (*k* = 0.154 nm) radiation with a generator voltage of 40 kV and a current of 40 mA. The scanning speed was 5° min^−1^ and the step size was 0.02° with the range from 10° to 60°. The surface and cross-section morphology of samples were observed by scanning electron microscopy (SEM, FEI Quanta FEG, USA) with an accelerating voltage of 20 kV. The elemental distribution mapping of Gr-PANI_10:1_ was measured by energy dispersive spectrometer (EDS, Oxford Instruments, United Kingdom). The electrical conductivity of films was measured by in-line four-point probe method (RTS-9, Scientific Equipment and Services) at room temperature, and three specimens with the thicknesses of 0.04 mm were used for each sample. The tensile strength and the elongation at break of the Gr-PANI@PI and Gr@PI films were measured by a universal stretching machine (UTM, 5567A, Instron, USA). Specimens were tested at a speed of 2 mm min^−1^ and a preload of 0.1 N. The size of each sample was 10 mm in length and 4 mm in width. Five specimens were used for each sample in the tensile test. The EMI shielding properties of the composite films were performed in the frequency range of 8.2–12.4 GHz (X band) using the waveguide method *via* the vector network analyzer (Agilent N5222B). All samples were cut into rectangle plates with a size of 22.8 × 10.1 mm^2^ to fit the waveguide sample holder. The scattering parameters (*S*_11_ and *S*_21_) of the Gr-PANI@PI composite films were collected to calculate the EMI shielding effectiveness. Thermogravimetric analysis (TGA, Q5000, TA Instruments, USA) was performed to determine the thermal stability of films. Thermal gravimetric analysis (TGA) was conducted under the N_2_ atmosphere from 50 to 900 °C at a heating rate of 10 °C min^−1^.

## 3. Results and discussion

### 3.1. Characteristics and morphology

**Fig. 1:**
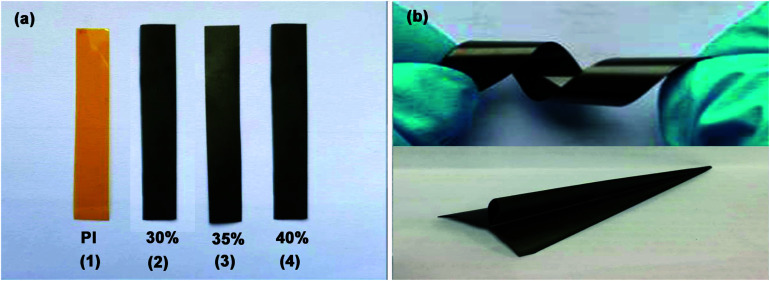
(a) The photos of pure PI and PI films with different Gr-PANI_10:1_ contents; (b) the flexible 40%Gr-PANI_10:1_@PI film.

The optical photos of PI and Gr-PANI_10:1_@PI film with different loading of 30%, 35% and 40% are displayed in Fig. 1(a). With the increasing amount of the filler, the Gr-PANI_10:1_@PI films become darker compared to the transparent yellow PI film. Furthermore, the 40%Gr-PANI_10:1_@PI still remains excellent flexibility. It can be twisted and folded into a paper plane (Fig. 1(b)). The size of 40%Gr-PANI_10:1_@PI film reaches 22 × 33 cm^2^ as displayed in Fig. S1(a) in ESI.† The dispersibility of filler in a polymer matrix is crucial for the preparation of high performance composites.^15^ In our experiment, the PANI is *in situ* synthesized on the surface of Gr sheets to connect adjacent Gr sheets through π–π non-covalent bonding. When the Gr-PANI composites are added into the NMP, the PANI can effectively prevent Gr sheets from agglomerating.^16,17^ As displayed in Fig. S1(b) in ESI,† after 8 hours of standing, Gr sheets aggregate partly in NMP, but, Gr-PANI_10:1_ composite filler shows excellent dispersibility due to the π–π non-covalent interactions between Gr and PANI, indicating that the PANI can avoid the agglomeration of Gr sheets in the organic solvent.^18^

**Fig. 2:**
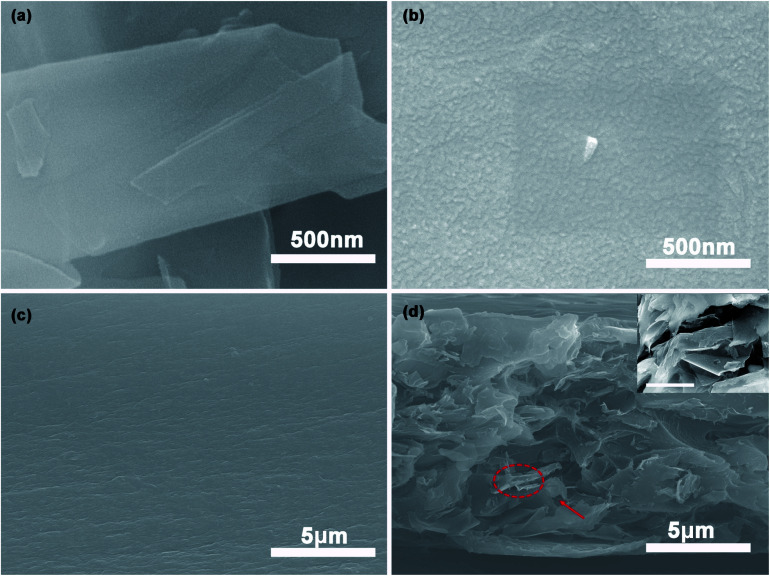
The SEM images of (a and b) surface morphology of pure Gr, Gr-PANI_10:1_; the cross-section images (c and d) of pure PI film, 40%Gr-PANI_10:1_@PI film. The inset shows the magnification of the selected area and the scale bar is 0.5 μm.

The SEM morphologies of Gr, Gr-PANI_10:1_, PI and 40%Gr-PANI_10:1_@PI are shown in Fig. 2. The pure Gr (Fig. 2(a)) displays a relatively smooth and flat surface. However, in Fig. 2(b), there are some particles attached on the surface of Gr sheets. It demonstrates that the PANI is aligned on the Gr surface homogeneously, which is ascribed to the π–π interaction between PANI and Gr.^19,20^ The size of the PANI absorbed on the Gr sheets is smaller than that of pure PANI (Fig. S3(a) in ESI†), which is beneficial for shortening the diffusion path of electrons and for enhancing the conductivity of the composite film.^21^ Additionally, the EDS mapping of Gr-PANI_10:1_, as demonstrated in Fig. S2,† shows that the C and N element is uniformly distributed on the Gr-PANI_10:1_ filler. The atomic percentage of N reaches 22.19 at%, indicating the successful attachment of PANI in the filler (Table S1†). While in the Gr-PANI_5:1_ (Fig. S3(b) in ESI†), the agglomeration of PANI can be observed on the surface of the Gr sheets, hampering the formation of the conductive network. The results indicate that the excessive PANI will lead to agglomeration in the composites. The cross sections of the pure PI and the 40%Gr-PANI_10:1_@PI are further investigated, as shown in Fig. 2(c) and (d). The PI film displays a relatively smooth structure, while the cross-section of Gr-PANI_10:1_@PI exhibits a typical stake-up morphology, demonstrating that Gr sheets are uniformly distributed in the PI matrix and form coarser fractured surfaces compared with pure PI. It can be attributed to the excellent compatibility and interfacial interaction between the Gr-PANI_10:1_ composites and the PI matrix.^22^ The PANI acts as the solder to connect adjacent Gr sheets into an integrated conductive structure with less gaps, thereby improving the electrical performance.

**Fig. 3:**
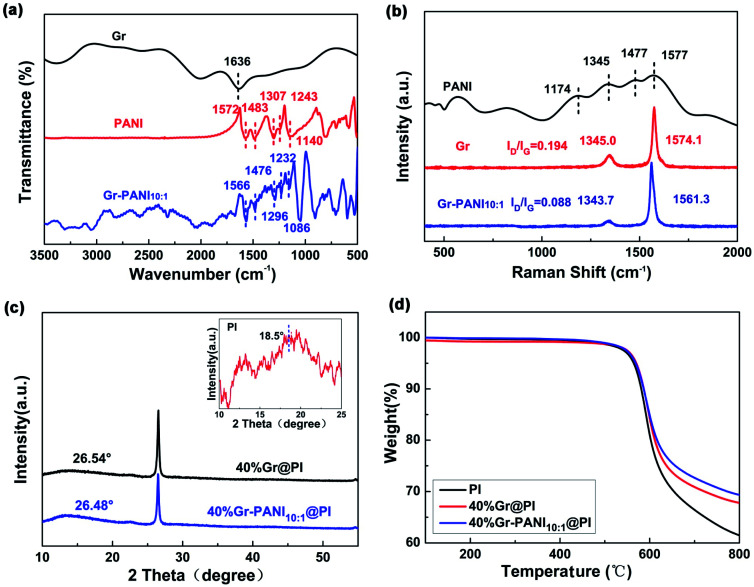
(a) FTIR spectra of pure Gr, PANI and Gr-PANI_10:1_ composites; (b) Raman spectra of Gr and Gr-PANI_10:1_ composites (excitation with 532 nm); (c) the XRD patterns of Gr@PI and Gr-PANI_10:1_@PI (the inset shows the XRD pattern of pure PI); (d) TGA curves of pure PI, Gr@PI and Gr-PANI_10:1_@PI films.

Fig. 3(a) shows the FTIR spectra of Gr, PANI, and Gr-PANI_10:1_ filler. The typical adsorption peaks of PANI are located at 1572 cm^−1^, 1483 cm^−1^, 1307 cm^−1^, 1243 cm^−1^ and 1140 cm^−1^, which represent C=C vibration in the quinoid ring, C=C stretching in the benzene ring, C–N stretching in the secondary aromatic amines, and C–H bonding in the benzenoid ring and the quinoid ring,^23^ respectively. In the Gr-PANI_10:1_, these characteristic peaks are shifted to 1566 cm^−1^, 1476 cm^−1^, 1296 cm^−1^, 1232 cm^−1^, and 1086 cm^−1^, respectively, indicating that PANI is distributed intimately on the surface of the Gr sheets,^24^ due to the electrostatic interactions of π–π non-covalent bonding between the Gr and PANI.^25^ The peak shift can be also observed in Gr-PANI_15:1_ and Gr-PANI_5:1_ (Fig. S4(a) (ESI†)).

To investigate the interaction between Gr sheets and PANI, the Raman spectroscopy of PANI, Gr and Gr-PANI_10:1_ is conducted, as displayed in Fig. 3(b). There are two prominent peaks corresponding to the G-band (∼1570 cm^−1^) of the first-order scattering of E_2g_ mode of sp^2^-hybridized carbon atoms and the D-band (∼1345 cm^−1^) of the disordered amorphous carbon C–C bonds at the defects or edge boundaries,^26^ respectively. After the *in situ* polymerization of PANI, the G-band and the D-band of the Gr-PANI_10:1_ are shifted to 1561.3 cm^−1^ (*vs.* 1574.1 cm^−1^ for Gr), and 1343.7 cm^−1^ (*vs.* 1345.0 cm^−1^ for Gr), respectively, suggesting the π–π interaction between PANI and Gr sheets.^27^ Moreover, the *I*_D_/*I*_G_ values of Gr and Gr-PANI_10:1_ are 0.194 and 0.088, respectively. With the decreasing mass ratio of Gr to PANI, the decrease in *I*_D_/*I*_G_ values can be also observed in Gr-PANI_15:1_ and Gr-PANI_5:1_ (Fig. S4(b) (ESI†)). The intensity ratio of D to G bands in Gr-PANI composites is attributed to the intimate interaction between the PANI and Gr sheets.^23,28,29^ Together with the FTIR spectra, the Raman results confirm that the Gr sheets are welded by PANI through π–π bonding during the *in situ* polymerization, thereby obtaining the PI film with high electrical property.

Fig. 3(c) exhibits the XRD patterns of pure PI, 40%Gr@PI and 40%Gr-PANI_10:1_@PI. The XRD pattern of pure PI shows a broad peak centered at 2*θ* = 18.5°.^30,31^ The sharp peaks centered at ∼26.50° in 40%Gr@PI and 40%Gr-PANI_10:1_@PI are attributed to the (002) of Gr. The 40%Gr@PI shows an obvious diffraction peak at 2*θ* = 26.54° corresponding to the *d*_(002)_ of 0.3362 nm, revealing the incorporation of PANI enlarges the interlayer space of graphene.^30^

The thermal stability is a key property of PI-based composite. Fig. S5† shows that the TGA curves of pure PANI, Gr and Gr-PANI_10:1_ composites under nitrogen atmosphere. The 15 wt% weight loss temperature of Gr-PANI_10:1_ shifts to higher temperature compared with pure PANI and Gr. The thermal stability of Gr-PANI_10:1_ is remarkably enhanced due to the presence of PANI and the π–π non-covalent between the Gr sheets and PANI.^12^ Meanwhile, TGA results demonstrate that the as-fabricated Gr-PANI_10:1_ composites contain 12.5 wt% of PANI by calculation. Fig. 3(d) presents the TGA curves of pure PI, 40%Gr@PI, and 40%Gr-PANI_10:1_@PI at a heating rate of 10 °C min^−1^ in nitrogen atmosphere. The pure PI film shows an onset weight loss below 100 °C due to the vaporization of water.^32^ The main mass loss occurs at approximately 500 °C, which is attributed to thermal decomposition of PI.^33^ The 15% weight loss temperatures of pure PI, the 40%Gr@PI, and 40%Gr-PANI_10:1_@PI are 592 °C, 599 °C and 600 °C, respectively. Meanwhile, the residual weight at 700 °C for the 40%Gr-PANI_10:1_@PI is evidently improved from 66.4% (pure PI) to 72.7%, indicating that the Gr-PANI_10:1_@PI film shows superior thermal stability. This phenomenon is owing to the excellent thermal stability of Gr-PANI_10:1_, its introduction reduces the TGA rate of the PI film.^34^

**Fig. 4:**
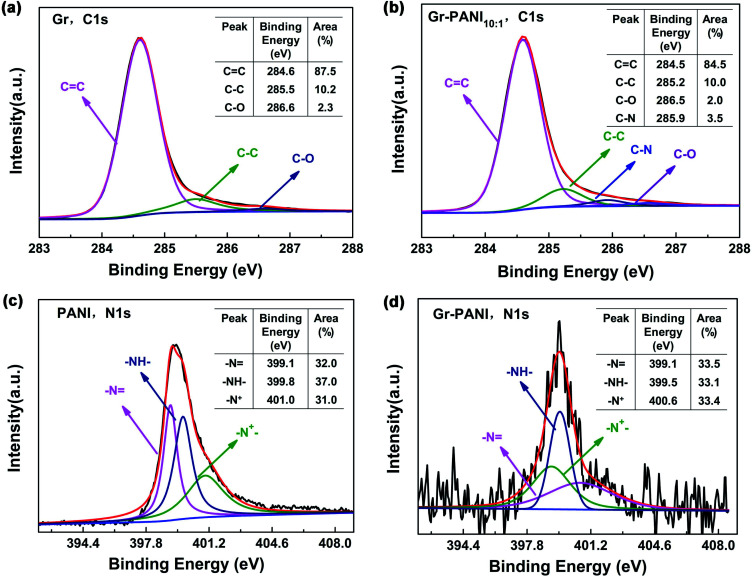
XPS spectrum of (a) and (b) C 1s spectrum of Gr and Gr-PANI_10:1_; (c) and (d) N 1s spectrum of PANI and Gr-PANI_10:1_.

XPS spectroscopy is further characterized to analyze the elemental compositions and the valence states of Gr, PANI and Gr-PANI_10:1_. The C 1s spectrum of Gr (Fig. 4(a)) consists of three peaks occurring at 284.6 eV, 285.5 eV and 286.6 eV, corresponding to the C=C, C–C and C–O, respectively.^35^ The content of each bonding is 87.5 atom%, 10.2 atom%, and 2.3 atom%, respectively. The three peaks of Gr-PANI_10:1_, as shown in the Fig. 4(b),are shifted to 284.5 eV, 285.2 eV and 286.5 eV, which is ascribed to the π–π interaction between Gr and PANI.^36^ After the *in situ* polymerization of PANI, the three peak areas show negligible changes 84.5 atom% (*vs.* 87.5 atom% for Gr), 10.0 atom% (*vs.* 10.2 atom% for Gr), 2.0 atom% (*vs.* 2.3 atom% for Gr), suggesting the interaction between Gr and PANI is not covalent bonding. Furthermore, an additional peak at 285.9 eV (3.5 atom%) corresponding to the C–N bonding is observed, suggesting that PANI has been successfully *in situ* synthesized on Gr sheets. The core-level N 1s spectrum of pure PANI presents three primary peaks –N= at 399.1 eV, –NH_2_ at 399.8 eV and –N^+^ at 401.0 eV in Fig. 4(c).^37^ The peak shift is also observed in Gr-PANI_10:1_ (Fig. 4(d)), and the three peaks move toward lower binding energy values of 399.1 eV, 399.5 eV and 400.6 eV, respectively.^38^ Furthermore, there are no obvious changes in three peak areas 33.5 atom% (*vs.* 32.0 atom% for PANI), 33.1 atom% (*vs.* 37.0 atom% for PANI), 33.4 atom% (*vs.* 31.0 atom% for PANI).The above results confirm the gap between the Gr sheets could be coupled together by PANI *via* the π–π non-covalent bonding, which can significantly enhance the dispersibility of Gr sheets.

### 3.2. Electrical conductivity

The electrical conductivity (*σ*) of each filler is measured by four-point probe method (RTS-9, Scientific Equipment and Services). The pure Gr has the highest *σ* of 618.1 ± 46.2 S cm^−1^ compared with Gr-PANI composites, as displayed in Fig. S6.† With the increasing amount of PANI, the *σ* decreases to 541.0 ± 16.9 S cm^−1^, 490.3 ± 39.0 S cm^−1^, 392.4 ± 44.5 S cm^−1^, corresponding to Gr-PANI_15:1_, Gr-PANI_10:1_ and Gr-PANI_5:1_, respectively. Such a decrease in *σ* is primary attributed to the factor that the electrical property of PANI is inferior to that of Gr. Furthermore, the *σ* of the 40%Gr@PI is only 1.4 ± 0.1 S cm^−1^ due to the inevitable agglomeration of Gr sheets in PI matrix. But with the increasing mass ratio of Gr to PANI from 15 : 1 to 10 : 1 at a filler loading of 40%, the *σ* increases to 1.8 ± 0.2 S cm^−1^ and 2.1 ± 0.1 S cm^−1^, but when the ratio is up to 5 : 1, the *σ* displays a dramatic decline to 1.3 ± 0.1 S cm^−1^. It is noticeable that the maximum *σ* reaches 2.1 ± 0.1 S cm^−1^ for 40%Gr-PANI_10:1_@PI, indicating the PANI welds the adjacent Gr sheets into conductive network so that it can increase the efficiency of electron transfer in PI film *via* the “non-covalent welding” strategy. However, with the further addition of the PANI (Gr-PANI_5:1_), there is an obvious decrease of *σ* in 40%Gr-PANI_5:1_@PI. Taking the result of SEM images into consideration, the reason lies in that the surface of Gr sheets is wholly covered by excessive PANI. The excessive PANI solder would lead to agglomeration between the Gr sheets, impeding the electron transfer.

We also make a comparison between the Gr@PI and the Gr-PANI_10:1_@PI with different filler contents, as shown in Fig. S6(b).† With the increasing amount of Gr from 30% to 40%, the *σ* improves from 0.4 ± 0.1 S cm^−1^ to 1.4 ± 0.1 S cm^−1^, respectively. In contrast, with the introduction of PANI, the *σ* of PI is improved further. When the filler content is at 40%, the *σ* of Gr-PANI_10:1_@PI is up to 2.1 ± 0.1 S cm^−1^, which is approximately 45% higher than that of Gr@PI film. The results demonstrate that the “non-covalent welding” strategy can successfully weld up the Gr sheets to improve the dispersibility of Gr and form conductive network in PI matrix, which can significantly enhance the *σ* of the composite film.

### 3.3. Mechanical properties

The mechanical properties and stress–strain curves of the Gr@PI and Gr-PANI_10:1_@PI are shown in Fig. S7 and S8 (ESI†), respectively. The tensile strength decreases gradually with the increasing amount of filler from 65.9 ± 1.0 MPa (30%) to 54.3 ± 2.8 MPa (40%). Furthermore, the tensile strength of the Gr-PANI_10:1_@PI is higher than that of Gr@PI with identical filler loadings. Compared with 40%Gr@PI (54.3 ± 2.8 MPa), the tensile strength of 40%Gr-PANI_10:1_@PI increases to 56.8 ± 1.4 MPa. The elongation at break of Gr@PI and Gr-PANI_10:1_@PI presents a slight decline with increasing filler contents (Fig. S7(b)†). The elongation at break of the 30%Gr@PI is only 3.4 ± 0.3%. However, the 30%Gr-PANI_10:1_@PI displays a slight increase in the elongation at break, which is about 1.29 times higher than that of 30%Gr@PI. The improvement in mechanical properties can be primarily ascribed to the homogeneous dispersibility of Gr-PANI_10:1_ in PI matrix because of the “non-covalent welding” strategy.^39^

### 3.4. EMI shielding effectiveness

The total EMI shielding effectiveness (SE_T_) of the Gr@PI and Gr-PANI_10:1_@PI is measured with vector network analyzer at the X-band (Fig. S9 (ESI†)). The total EMI SE_T_ of shielding materials can be defined as the logarithmic of the ratio of the incident wave *P*_I_ to the transmitted wave *P*_T_, which is calculated by the following equation:^40^1
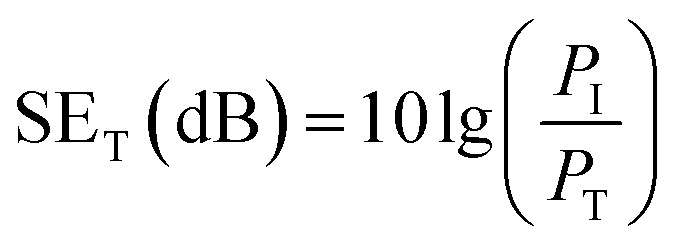


In general, the electromagnetic wave consists of three aspects: reflection (SE_R_), absorption (SE_A_) and multiple reflections (SE_M_), respectively. The EMI performance is attributed to the interaction between the electromagnetic waves and free electrons on the surface of materials. The material with high electrical conductivity can provide electric dipoles. When the incident electromagnetic waves interact with the free electrons on the surface and some electromagnetic waves are reflected from the surface of the shield. If electric dipoles interact with the electromagnetic fields in the radiation, the remaining electromagnetic waves would be absorbed by the dipoles.^41–43^ In addition, the thickness also affects the value of SE_M_ and SE_A_. The SE_M_ can be neglected when the value of SE_T_ is larger than 10 dB, therefore, SE_T_ can be simplified as the equation:^44^

2 SE_T_ = SE_R_ + SE_A_

In a vector network analyzer, we can record the parameters (*S*_11_ and *S*_21_) to calculate the EMI SE as shown in following equation:^45^

3 *T* = |*S*_11_|^2^, *R* = |*S*_21_|^2^, *A* = 1 − *R* − *T*

4
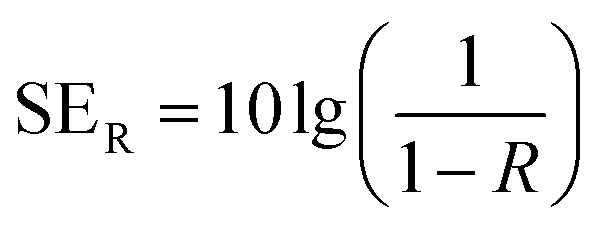
5
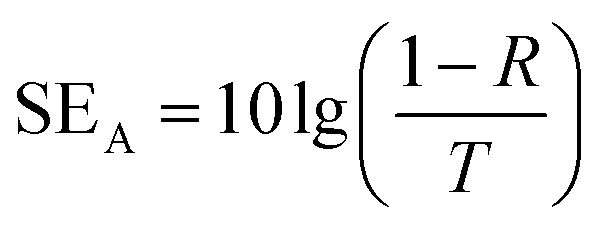


As illustrated in the equation, *T* is transmittance coefficient, *A* is absorbance coefficient, *R* is reflection coefficient.

**Fig. 5:**
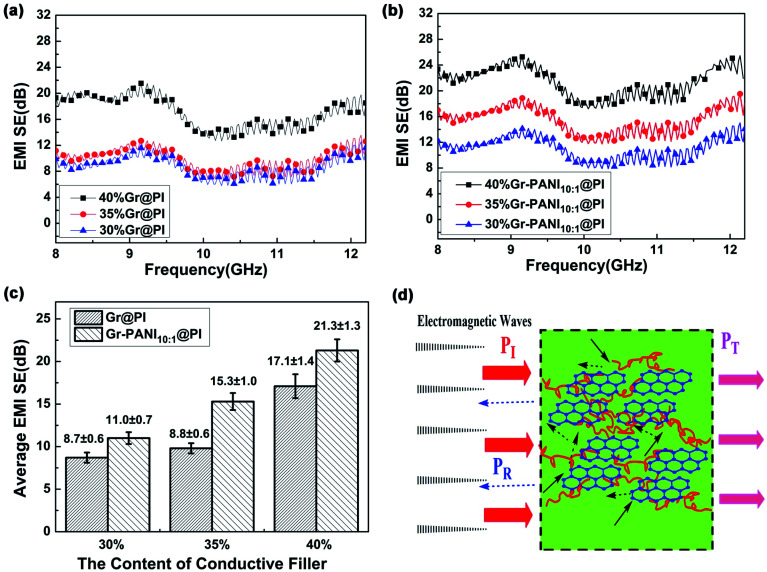
(a) SE_T_ of Gr@PI; (b) SE_T_ of Gr-PANI_10:1_@PI; (c) comparison of average SE_T_ of Gr@PI and Gr-PANI_10:1_@PI with different contents; (d) EMI shielding mechanism of Gr-PANI_10:1_@PI (*P*_I_: incident wave; *P*_R_: reflected wave; *P*_T_: transmitted wave).

The SE_T_ of the PI films with different amount of Gr and Gr-PANI_10:1_ fillers is shown in Fig. 5(a) and (b). The electrical property plays an important role in the SET of composite films. As the amount filler from 30% to 40%, the SE_T_ values of both Gr@PI and Gr-PANI_10:1_@PI increase significantly due to the increasing electrical conductivity. As shown in Fig. 5(c), the SE_T_ of Gr-PANI_10:1_@PI is ∼21.3 dB at 40%, which is approximately 125% enhancement compared with Gr@PI (∼17.1 dB). The uniformly dispersed Gr sheets could attenuate electromagnetic radiation easily due to reflection and scattering microwave in the Gr-PANI_10:1_@PI film, and incident electromagnetic microwaves are transferred to heat by being absorbed. The Gr-PANI_10:1_@PI shows higher SE_T_ value compared with Gr@PI, which can be ascribed to the structure of conductive network by the “non-covalent welding” approach.

The high electrical conductivity and the formation of conductive networks within the PI matrix play an important role in the EMI shielding performance of the film. Fig. 5(d) shows the EMI shielding mechanism of Gr-PANI_10:1_@PI film. A portion of electromagnetic wave *P*_I_ is reflected at the interface of the film, because conductive networks with a large amount of charge carriers are beneficial to EMI shielding effectiveness by reflection. The remaining portion penetrating the surface is multiply reflected or absorbed by the Gr layers.^46^ Only a small amount of electromagnetic waves pass through the film. The formation of an excellent conductive network between Gr and PANI in PI matrix ensures that the electromagnetic wave is greatly reduced, leading to an outstanding EMI shielding performance. On the other hand, the Gr-PANI@PI film possesses the continuously cross-linked network structure entirely consisted of Gr-PANI layers, which could certainly enhance internal multiple reflections more effectively than the agglomerated Gr layers in PI matrix. With the effect of the above two aspects, the EMI performance of Gr-PANI_10:1_@PI film has been obviously improved.^47,48^

Generally, the density and thickness of a material are also two significant factors for evaluating its EMI shielding performance. Hence, the SSE on the basis of the EMI SE_T_, the density, and the thickness is an appropriate criterion to estimate the shielding performance of various EMI shielding materials. The SE_T_ value of reported EMI shielding films in the X-band region is listed in Table S2.† Compared with other carbon-based polymer films, the 40%Gr-PANI@PI_10:1_ has more excellent SE_T_ (21.3 dB) and higher SSE (4096.2 dB cm^2^ g^−1^) with the thickness only of 0.04 mm. This value is higher than that of the PS/graphene (∼17.3 dB) prepared by high-pressure compression molding^49^ and PU/MWCNT (∼20.0 dB) prepared ball mill method,^50^ respectively. Moreover, it is also superior to that of the WPU/CNT composites by a facile freeze-drying method by 72.6% (∼2143.0 dB cm^2^ g^−1^)^51^ and PI/rGO (17–21 dB)^52^ with a thickness of 0.8 mm. The survey suggests that Gr-PANI@PI_10:1_ film has the potential advantages to meet the requirement of the commercial application (more than 20 dB).

### 3.5. The mechanism of “non-covalent welding”

**Scheme 2:**
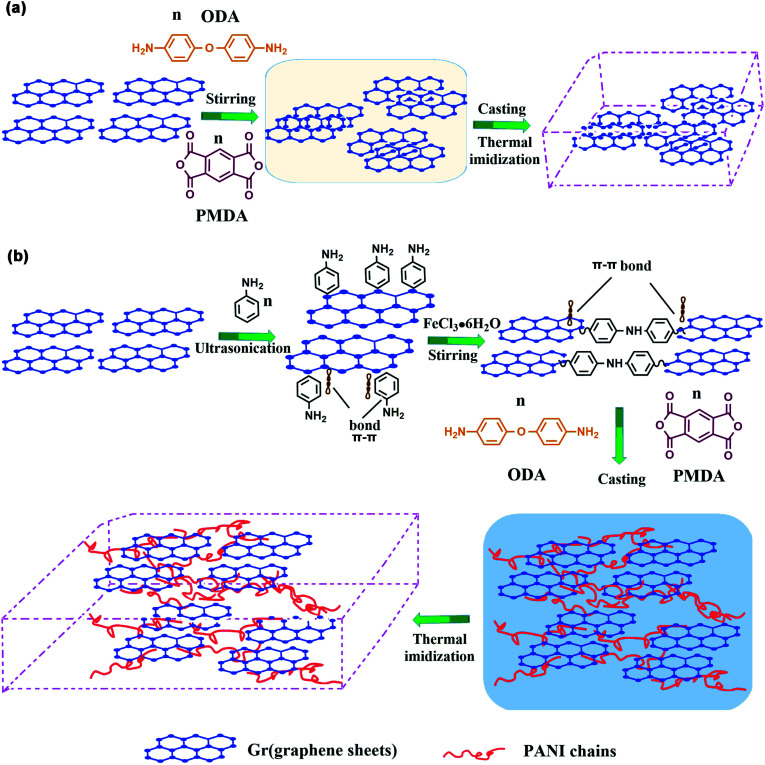
The mechanism of (a) Gr@PI without PANI and (b) Gr-PANI_10:1_@PI with PANI *via* “non-covalent welding”.

The mechanism of “non-covalent welding” between PANI and Gr is illustrated in Scheme 2. In Scheme 2(a), the Gr@PI film is directly fabricated by *in situ* polymerization. Without the PANI, during stirring process, the Gr sheets tend to agglomerate due to Vander Waals force and π–π stacking interaction between Gr sheets resulting in an inhomogeneous and discrete network.^53^ In Scheme 2(b), the PANI is *in situ* synthesized on the surface of Gr sheets to weld up adjacent Gr sheets through π–π non-covalent bonding, which effectively avoids the agglomeration of Gr sheets in organic solvent. After the thermal imidization, Gr-PANI forms a continuous conductive network in polymer matrix, making the electron transfer more effective. In this case, the combination of Gr and PANI only depends on π–π non-covalent bonding, and the structure of Gr is not destroyed, ensuring the excellent conductivity of Gr.

## 4. Conclusions

In summary, we have reported a novel “non-covalent welding” approach to prepare Gr-PANI_10:1_@PI film with enhanced EMI shielding performance and electrical properties. The PANI, as a solder, can successfully weld up the graphene sheets *via* π–π non-covalent bonding and form a continuous conductive network in polymer matrix by *in situ* polymerization. The Gr-PANI_10:1_@PI exhibited a superior SE_T_ of 21.3 dB and SSE of 4096.2 dB cm^2^ g^−1^ at 40% content with a thickness of 40 μm compared to that of the Gr@PI (SE_T_ 17.1 dB). The *σ* of Gr-PANI_10:1_@PI was promoted to 2.1 ± 0.1 S cm^−1^ higher than Gr@PI (1.4 ± 0.1 S cm^−1^) at 40% content. Last but not least, we believe that such a “non-covalent welding” strategy shows a great potential to fabricate composites with inorganic fillers.

## Conflicts of interest

There are no conflicts to declare.

## Supplementary Material

RA-010-C9RA08026K-s001
