# Supplementary material for: In situ polymerization of graphene-polyaniline@polyimide composite films with high EMI shielding and electrical properties
Source: RSC Adv. 2020 Jan 13;10(4):2368–77. doi: 10.1039/c9ra08026k (PMC9048709; doi:10.1039/c9ra08026k)
Supplement: RA-010-C9RA08026K-s001 [file RA-010-C9RA08026K-s001.pdf]

Electronic Supplementary Material (ESI) for RSC Advances.
This journal is © The Royal Society of Chemistry 2019

Electronic Supplementary Material (ESI) for RSC Advances.
This journal is © The Royal Society of Chemistry 2019

Supporting Information

In situ polymerization of graphene-polyaniline@ polyimide nanocomposite films with high EMI shielding and electrical properties

Kui Cheng,† Haoliang Li,†* Mohan Zhu, Hanxun Qiu and Junhe Yang*
School of Material Science and Engineering, University of Shanghai for Science and Technology, Shanghai 200093, China
Kui Cheng, Haoliang Li, contributed equally to this work
Corresponding authors: lihl1989@outlook.com jhyang@usst.edu.cn

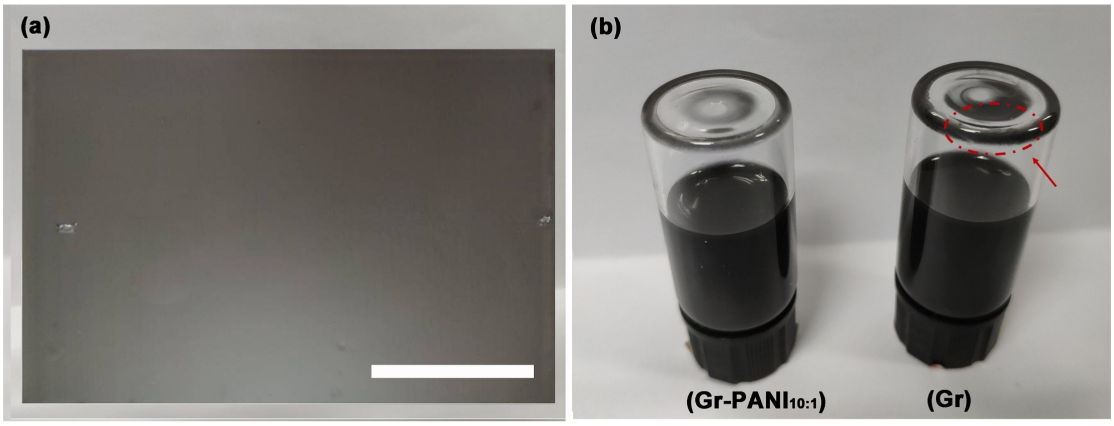

Fig. S1 (a) The large size 40%Gr-PANI$_{10:1}$@PI film (the scale bar is 10 cm); (b) Gr-PANI$_{10:1}$ in NMP and Gr in NMP.

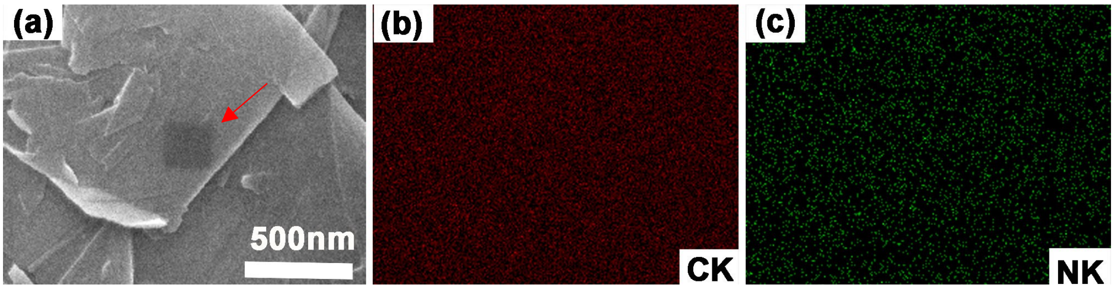

Fig. S2. SEM image of Gr-PANI$_{10:1}$ and the EDS mapping of Gr-PANI$_{10:1}$.

Table S1 Element content of Gr-PANI$_{10:1}$ composites.

Element (Gr-PANI$_{10:1}$)	At%
CK	77.15
NK	22.19
OK	00.66

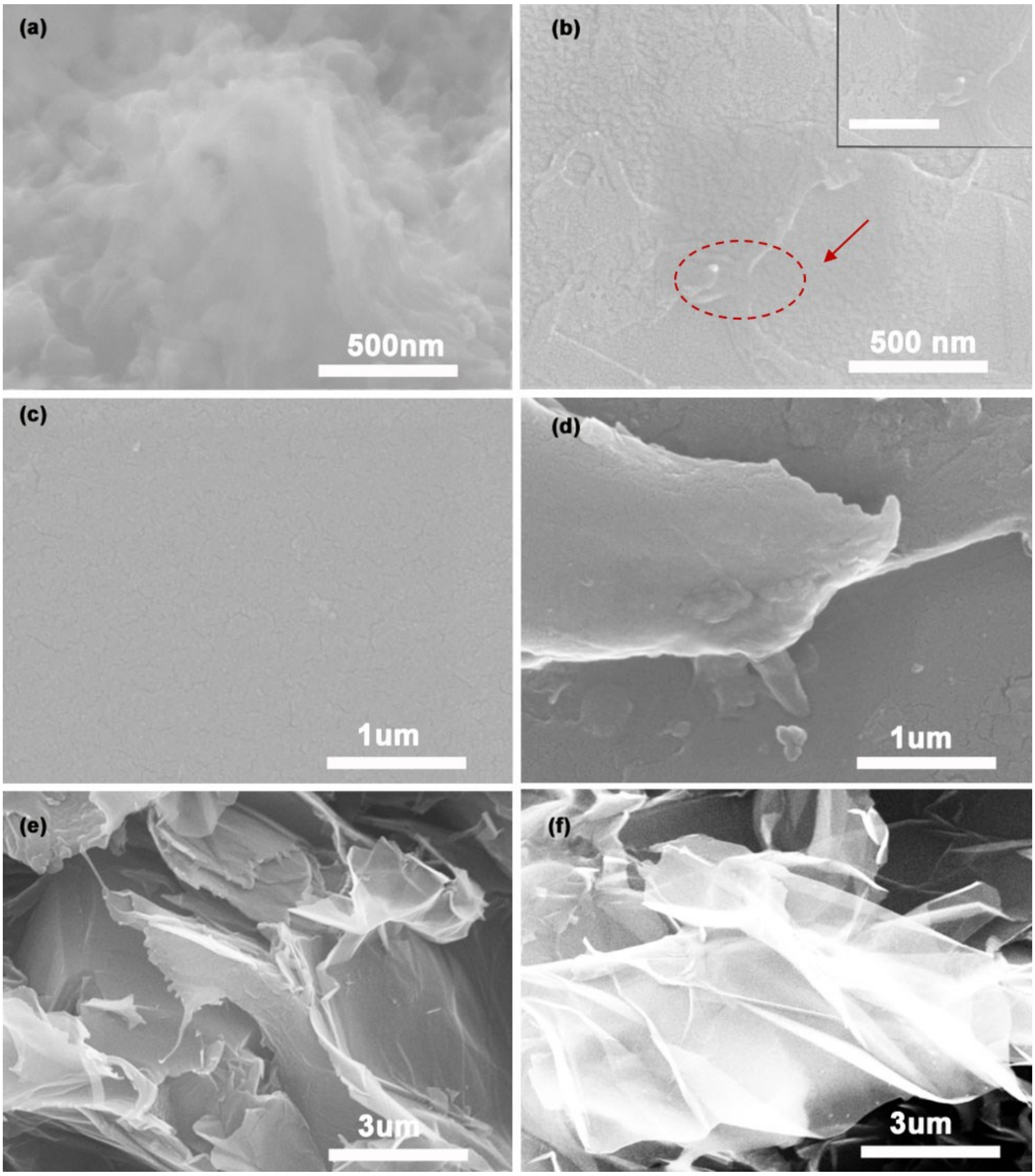

Fig. S3 The SEM images of (a, b,c,d) surface morphology of PANI, Gr-$PANI_{5:1}$,Gr-$PANI_{10:1}$@PI and PI; The cross-section images (e, f) of Gr@PI and Gr-$PANI_{10:1}$@PI; The inset shows the magnification of the selected area and the scale bar is 0.1 um.

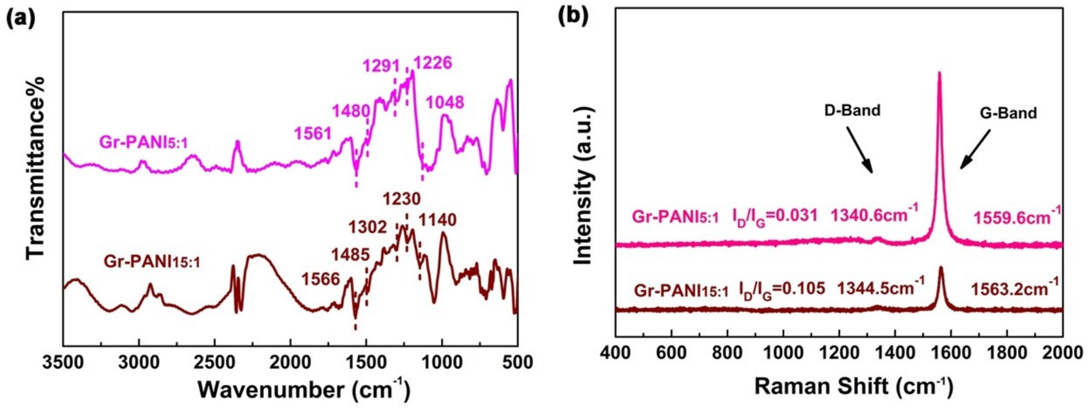

Fig. S4 (a) FTIR spectra of Gr-PANI$_{5:1}$ and Gr-PANI$_{15:1}$ composites; (b) Raman spectra of Gr-PANI$_{5:1}$ and Gr-PANI$_{15:1}$ composites.

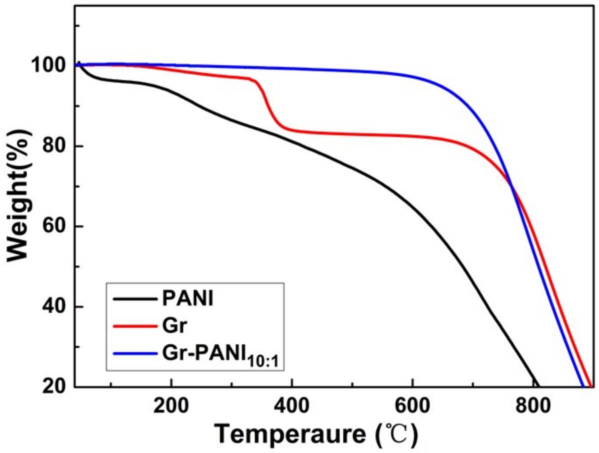

Fig. S5 TGA curves of pure PANI, Gr and Gr-PANI$_{10:1}$ composites.

Fig. S5 shows TGA curves of pure PANI, Gr and Gr-PANI$_{10:1}$ composites. The 15 wt% weight loss temperatures of the PANI, Gr and Gr-PANI$_{10:1}$ are 328.5 °C, 383.1 ℃, and 717.5℃, respectively. Meanwhile, the residual weight at 700 ℃ for the Gr-PANI$_{10:1}$ is remarkably enhanced from 79.3% (pure Gr) to 86.8%, indicating that the Gr-PANI$_{10:1}$ shows superior thermal stability. The TGA curve of Gr-PANI$_{10:1}$ is the superposition of Gr and PANI. The weight ratio of PANI in Gr-PANI$_{10:1}$ is calculated by the following equation (1):

$$\text{wt\%(PANI)} = 1 - \frac{\Delta W2 - \Delta W1}{\Delta W2 - \Delta W3} \times 100\% \quad (1)$$

where the ΔW_1, ΔW_2 and ΔW_3 represent the weight loss of Gr-PANI$_{10:1}$, PANI and Gr from 40 to 800 °C. According to the TGA data, the PANI content in Gr-PANI$_{10:1}$ is 12.5 wt%, which is close to the theoretical value.

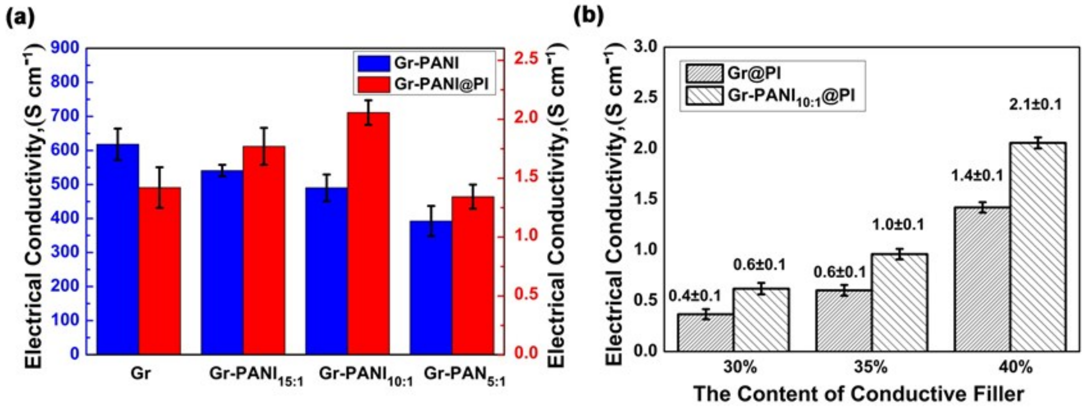

Fig. S6 Electrical conductivity of (a) Gr-PANI and 40%Gr-PANI@PI with different mass ratios of Gr to PANI; (b) Gr@PI and Gr-PANI$_{10:1}$@PI with different filler contents.

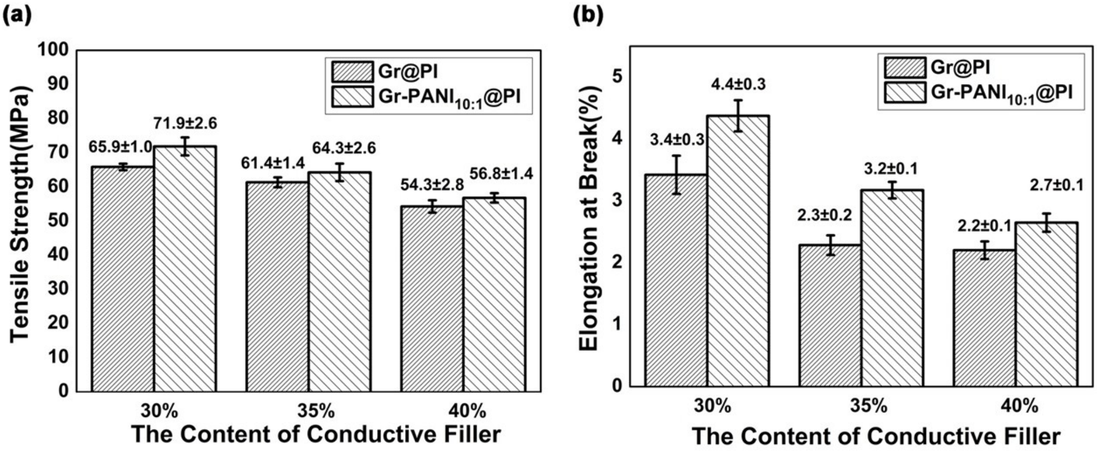

Fig. S7 The mechanical properties of Gr@PI and Gr-PANI$_{10:1}$@PI with different filler contents (a) the tensile strength; (b)the elongation at break.

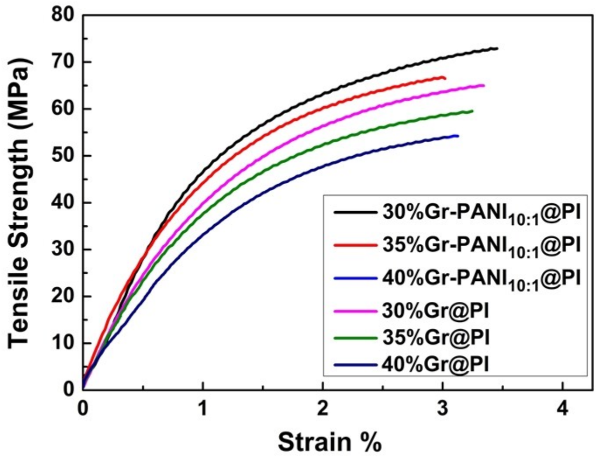

Fig. S8 Typical stress–strain curves of pure Gr@PI and Gr-PANI$_{10:1}$@PI films with different filler contents.

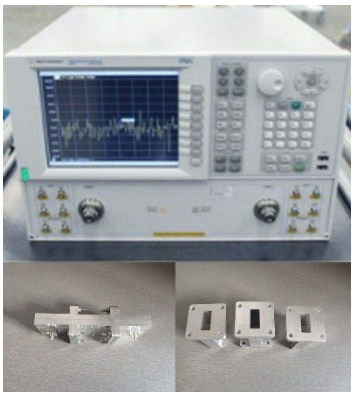

Fig. S9 Experiment setup for EMI shielding measurement: vector network analyzer.

Table S2 EMI shielding performance of various carbon-based polymer composite films

Materials	Content (wt%)	Thickness (mm)	EMI SE_T (dB)	EMI SSE value ($dB \cdot cm^2\ g^{-1}$)	Ref
PS/graphene	30.0	2.50	~17.3	~256.3	[1]
PEI/graphene foam	10.0	2 .30	~11.0	~164.9	[2]
PU/MWCNT	22.0	0.10	~20.0	/	[3]
WPU/CNT	76.2	0.32	~49.0	~3408.0	[4]
PVDF/graphene	15.0	0.10	/	~1265.0	[5]
Phenolic/rGO	70.0	0.30	~43.4	/	[6]
PI/graphene	8.0	0.50	13.7–14.9	~693.0	[7]
PI/graphene foam	16.0	0.80	17.0-21.0	~`75.0	[8]
PE/graphene	15.0	1.00	~33.0	/	[9]
Cellulose/CNT	40.0	0.15	~35.0	~1372.4	[10]
WPU/CNT	76.2	2.30	~35.0	~2143.0	[11]
Gr-$PANI_{10:1}$@PI	40.0	0.04	~21.3	~4096.2	This work

References

1 D. X. Yan, P. G. Ren, H. Pang, Q. Fu, M. B. Yang and Z. M. Li, *J. Mater. Chem.*, 2012, **22**, 18772-18774.
2 J. Ling, W. Zhai, W. Feng, B. Shen and G. Z. Wen, *ACS Appl. Mater. Interfaces.*, 2013, **5**, 2677-2684.
3 A. S. Hoang, *Adv. Nat. Sci-Nano.*, 2011, **2**, 5766–5771.
4 Z. Zeng, M. Chen, J. Hao, W. Li, X. Xiao, L. Zhou, Y. Pei, Z. Hui and Z. Zhong, *Carbon.*, 2016, **96**, 768-777.
5 H. B. Zhang, Q. Yan, W. G. Zheng, Z. He and Z. Z. Yu, *ACS Appl. Mater. Interfaces.*, 2011, **3**, 918-924.
6 A. P. Singh, P. Garg, F. Alam, K. Singh, R. B. Mathur, R. P. Tandon, A. Chandra and S. K. Dhawan, *Carbon.*, 2012, **50**, 3868-3875.
7 H. Yang, Z. Li, H. Zou and P. Liu, *Polym. Adv. Technol.*, 2016,**28**(2),233-242.
8 Y. Li, X. Pei, B. Shen, W. Zhai, L. Zhang and W. Zheng, *RSC Adv.*, 2015, **5**(31), 24342-24351.
9 M. H. Al-Saleh, *J. Phys. D-Appl. Phys.*, 2016, **49**(19), 195302.
10 L.-Q. Zhang, B. Yang, J. Teng, J. Lei, D.-X. Yan, G.-J. Zhong and Z.-M. Li, *J. Mater. Chem. C.*, 2017, **5**, 3130-3138.
11 Z. Zeng, H. Jin, M. Chen, W. Li, L. Zhou and Z. Zhang, *Adv. Funct. Mater.*, 2016, **26**, 303-310.
